# Multidimensional Clinical Phenotyping of an Adult Cystic Fibrosis Patient Population

**DOI:** 10.1371/journal.pone.0122705

**Published:** 2015-03-30

**Authors:** Douglas J. Conrad, Barbara A. Bailey

**Affiliations:** 1 Department of Medicine, Division of Pulmonary, Critical Care and Sleep Medicine, University of California San Diego, La Jolla, California, United States of America; 2 Department of Mathematics and Statistics, San Diego State University, San Diego, California, United States of America; University of Alabama-Birmingham, UNITED STATES

## Abstract

**Background:**

Cystic Fibrosis (CF) is a multi-systemic disease resulting from mutations in the Cystic Fibrosis Transmembrane Regulator (CFTR) gene and has major manifestations in the sino-pulmonary, and gastro-intestinal tracts. Clinical phenotypes were generated using 26 common clinical variables to generate classes that overlapped quantiles of lung function and were based on multiple aspects of CF systemic disease.

**Methods:**

The variables included age, gender, CFTR mutations, FEV1% predicted, FVC% predicted, height, weight, Brasfield chest xray score, pancreatic sufficiency status and clinical microbiology results. Complete datasets were compiled on 211 subjects. Phenotypes were identified using a proximity matrix generated by the unsupervised Random Forests algorithm and subsequent clustering by the Partitioning around Medoids (PAM) algorithm. The final phenotypic classes were then characterized and compared to a similar dataset obtained three years earlier.

**Findings:**

Clinical phenotypes were identified using a clustering strategy that generated four and five phenotypes. Each strategy identified 1) a low lung health scores phenotype, 2) a younger, well-nourished, male-dominated class, 3) various high lung health score phenotypes that varied in terms of age, gender and nutritional status. This multidimensional clinical phenotyping strategy identified classes with expected microbiology results and low risk clinical phenotypes with pancreatic sufficiency.

**Interpretation:**

This study demonstrated regional adult CF clinical phenotypes using non-parametric, continuous, ordinal and categorical data with a minimal amount of subjective data to identify clinically relevant phenotypes. These studies identified the relative stability of the phenotypes, demonstrated specific phenotypes consistent with published findings and identified others needing further study.

## Introduction

Cystic fibrosis is a multi-system disease with clinical manifestations in sweat glands, sinuses, lungs, pancreas, hepato-biliary tree, and the lower gastrointestinal tract. These manifestations result from mutations in the Cystic Fibrosis Transmembrane Regulator (CFTR) gene which cause mucus dysfunction above epithelial surfaces [1]. In sino-pulmonary tissue, the mucociliary clearance mechanism is impaired and results in chronic polymicrobial infections typically dominated by climax populations such as *Pseudomonas* spp. (PA), *Staphylococcus aureus*, and other fungal species which are inherently resistant to anti-microbial therapy [2]. These chronic airway infections incite innate and adaptive immune responses which cause most of the morbidity and mortality associated with CF. Furthermore, 80–85% of patients suffer from pancreatic insufficiency which can result in severe nutritional deficiency. Other typical manifestations include distal intestinal obstruction syndrome from the loss of the lubricating effects of colonic mucus as well as diabetes, hepatic cirrhosis, and bone disease [3].

Multi-variable scoring and classification systems are important for identifying patients or classes of patients who are at risk for disease progression or may have distinct response rates to specific therapies [4]. In addition, classes of patients i.e. clinical phenotypes are critical for identifying specific genomic risk factors and host immune responses [5]. Clinical phenotypes are also used to demonstrate the metagenomic and metabolomic adaptations of the microbial (bacterial, viral and fungal) communities to the remodeled airway microenvironments and the resultant regional host responses [6,7,8].

The current study was initiated to generate phenotypes based on common clinical data with distinct gender, age, nutritional status and lung function risk characteristics. The phenotypes were generated from a recent data set and compared to an older one compiled three years earlier. The clinical variables in the analysis represent many of the potentially interacting sub-phenotypes of this multi-system disease and generated complex phenotypes with characteristics consistent with published phenotypes.

## Methods

### Recruitment

Every patient provided written informed consent and all data was obtained as part of their normal clinical care. For patients who died or had lung transplants, the data used was obtained during the 365 day period prior to the date of death or transplant.

**Fig 1:**
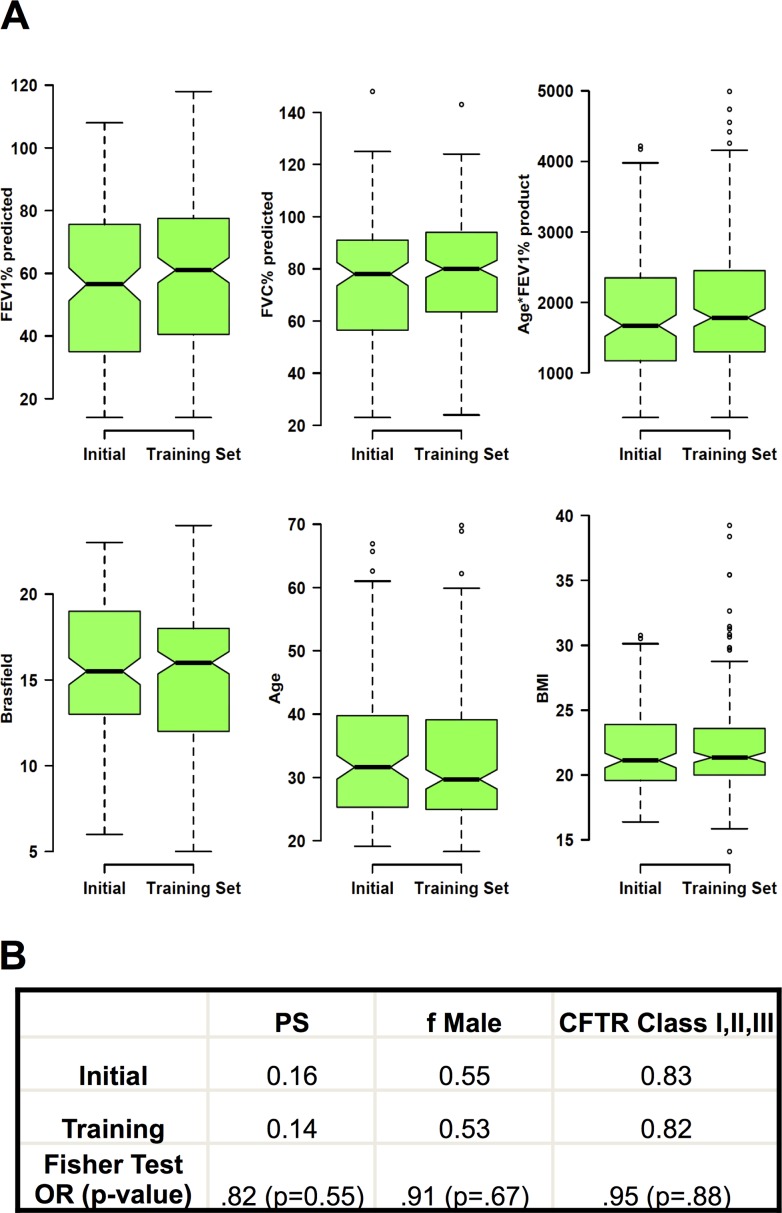
Comparison of the 2011 and 2014 adult CF patient cohorts. (A) Notchplots comparing the FEV1% predicted, FVC% predicted, Age*FEV1% product, Brasfield chest Xray scores, age and BMI. (B) Proportions of pancreatic sufficiency (PS), fraction of male subjects (f Male) and presence of two CFTR Class I, II and III mutations. The Fisher exact test odds ratio (OR) with the p-value are presented. Notchplots demonstrate the median, 25 percentile, 75 percentile and outlier values. The extending bars demonstrate the span between 1.5 x interquartile range above and below the median. Nonoverlapping notched areas likely represent significant differences between two groups.

Two cohorts of patients were recruited for these studies. The initial cohort was recruited between 2006 and 2011 and the dataframe was last updated in July of 2011. Two subjects in this cohort refused consent and an additional twelve patients were excluded because of incomplete data leaving 148 subjects with complete datasets. A larger cohort (the training data set) was recruited between 2011 and July 2014. Of 234 unique CF patients evaluated in the clinic, 211 patients were 1) seen more than once during the 2010 through 2014 study period, 2) signed informed consent, 3) had complete datasets 4) and did not have a transplant. The two cohorts were not statistically different in terms of height, weight, body mass index (BMI), lung function, frequency of either pancreatic sufficiency or presence of two CFTR class I, II or III mutations (Fig. 1). The training cohort contained updated data of the 119 subjects present in the original cohort and data from 92 new patients.

### Data Collection

The following information was collected on each subject: age, gender, CFTR mutations, the patients best FEV1% and FVC% predicted (Crapo references) for the prior 12 months, pancreatic sufficiency status, height, weight, body mass index (BMI), sputum microbiology and Brasfield chest xray score. Pancreatic sufficiency (PS) status was determined clinically i.e. no need for pancreatic enzyme replacement therapy to maintain weight and minimize pancreatic malabsorption symptoms. Presence of *Pseudomonas* spp, Methicillin Resistant *Staphylococcus aureus* (MRSA), Methicillin Sensitive *Staphylococcus aureus* (MSSA), *Achromobactor* spp, *Burkoholderia* spp, *Stenotrophomonas* spp, and fungal/mycobacterial species were called present if they were identified in at least two sputum cultures for the prior 12 months. Brasfield scores were obtained from the most recent chest xray and interpreted by a single reader (DJC) to minimize reading inconsistencies [9]. A product of the FEV1% predicted and age was calculated and used as an accrued lung health score, an approach used earlier in a study of homozygotic delF508 patients [10]. For the purposes of these studies, patients with at least one CFTR class IV, V, and VI mutation were grouped together and compared to the group containing two CFTR class I, II, or III mutations. A third category i.e. “unknown” was used for compound heterozygotes with only one known CFTR mutation or subjects without CFTR genetic assessments.

### Analysis

For the analysis, the modern multivariate statistical learning method, Random Forests, was implemented [11]. Random Forests consists of a collection or ensemble of classification trees where each tree is grown with a different bootstrap sample of the original data. Each tree votes for a class and the majority rule is used for the final prediction. Since each tree is grown with a bootstrap sample of the data, there are out-of-sample data available to calculate misclassification error. The out-of-sample data can also be used to determine variable importance for each variable. This is done for each tree by randomly permuting each variable in the out-of-sample data and recording the prediction. For the ensemble of trees, the permuted predictions are compared with the unpermuted predictions and aggregated. The magnitude of the decrease in accuracy indicates the importance of that variable. The variable importance is used in dimension reduction by providing a ranking of the variables by their importance measure. Random Forests implicitly handles interactions through the hierachical structure. These interactions may be local, depending on the splitting rule, and have an effect on only a subset of the observations. It is useful to explicitly incorporate interactions effects when they are known or make scientific or biologic sense. The interaction will appear as a single variable in the importance measure.

Random Forests can be used in both supervised and unsupervised learning. In the supervised Random Forests, each subject will have a known class or grouping. Each tree in the ensemble is grown using a binary recursive partition algorithm with a bootstrap sample of the original data and is unpruned. In unsupervised Random Forests, the data is classified without a priori classification specifications. Synthetic classes are generated randomly and the trees are grown. Despite the synthetic classes, similar samples will end up in the same leaves of the trees due to each tree’s branching process. The proximity of samples can be measured and a proximity matrix is constructed. Settings included the creation of 1500 decision trees and the remaining default settings. Clusters or groups can be detected using clustering algorithms, such as Partitioning Around Medoids (PAM). PAM uses the dissimilarity matrix (1-proximity) in its class partitioning or clustering algorithm. (R, library:cluster) PAM is more robust to noise and outliers as compared to the more commonly used k-means algorithm. The data were used to make progressively finer classes using k = 3, k = 4, k = 5 and k = 6 groupings. The presented data uses the k = 4 and k = 5 clustering strategy because of the size of the two cohorts and the planned analysis.

**Fig 2:**
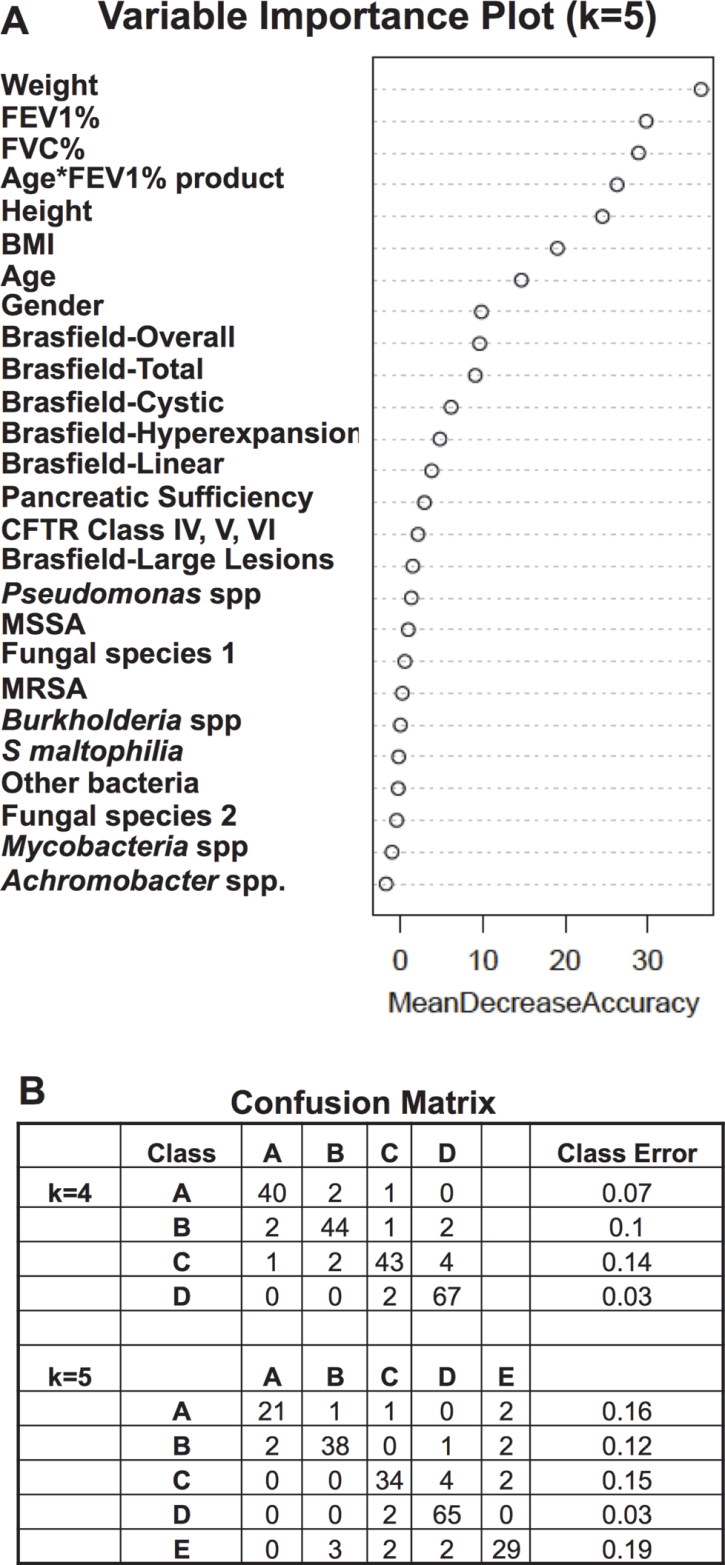
Characterization of the Adult CF Clinical Classes. (A) The variable importance plot generated by the supervised Random Forests algorithm of the classes derived from the proximity matrix of the unsupervised Random Forests (all variables) and the PAM clustering algorithm (k = 5 is shown). (B) The confusion matrix generated from the supervised Random Forests algorithm of the classes that were reformed after the described dimension reduction strategy. Overall out of bag error rates were 8.06% and 11.4% for the k = 4 and k = 5 classes, respectively.

**Fig 3:**
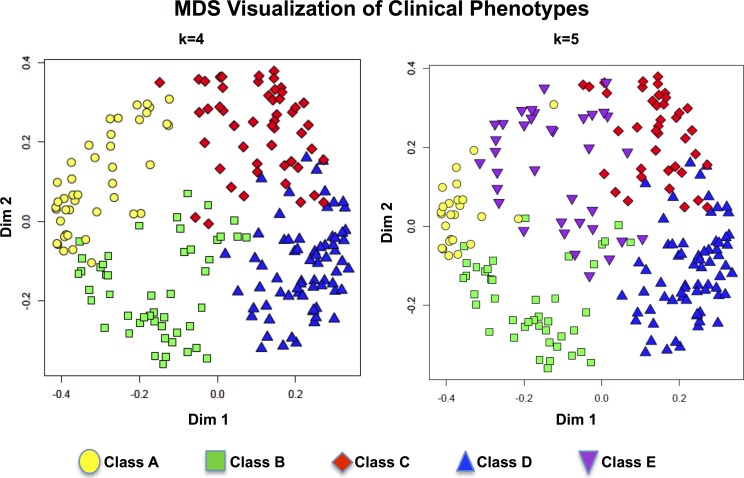
Visualization of the class separation (k = 4 and k = 5) using multidimensional scaling. (MDS) of the proximity matrix generated by the eight important clinical variables.

The analysis proceeded as follows:

1. The unsupervised Random Forest algorithm was used to generate a proximity matrix using all listed clinical variables.
2. PAM clustering of this first proximity matrix generated the initial classes.
3. A supervised Random Forest analysis of the initial classes a) indicated out of bag error rates of about 25–30%%. b) had variable importance plots demonstrating that FEV1%, FVC%, Age, Height, Weight, BMI, Age*FEV1% product and Brasfield scores were the most important variables in forming the classes. (Fig. 2A). This pattern was very similar with the k = 3, k = 4, and k = 6 classifications.
4. A dimension reduction strategy was used by repeating the unsupervised Random Forest analysis with these eight most important variables to generate a second proximity matrix.
5. PAM clustering was repeated using the second proximity matrix to generate the new classes which were further analyzed in the manuscript.
6. A supervised Random Forest analysis of the new classes demonstrated the lower out of bag error rates and the confusion matrix in Fig. 2B. MDS visualization of these new classes confirmed good separation of the classes using this dimension reduction strategy. (Fig. 3)

Clustering of particular traits such as pancreatic sufficiency, CFTR Class IV, V, and VI mutations, presence of *Pseudomona*s spp, *S*. *aureus* spp, and fungal species across the clinical phenotypes were assessed for significance using the Fisher exact test (two sided) using a significance threshold of p<.05. All p values were corrected for multiple comparisons using the method of Benjamini and Hochberg [12].

### Ethics Statement

All patients provided written informed consent (UCSD Human Research Protections Program applications #090159 or #081500) and all data was obtained as part of their normal clinical care. All clinical information was anonymized and deidentified to the extent possible and consistent with the approval of the institutional review board (UCSD Human Research Protections Program).

## Results

### Population Description

The subjects in both cohorts are a typical adult CF population in terms of demographics, distribution of CFTR mutations, nutritional parameters, lung function and prevalence of major CF pathogens [3]. There were no statistically significant differences in these parameters between the two cohorts or with excluded subjects. (Figs. 1 and S1)

Overall pulmonary health was estimated using the product of the patient’s most recent age with the highest FEV1% predicted in the prior 12 months. An earlier report using a similar approach in a homozygous ΔF508 CFTR CF patient population identified the borders dividing severe, moderate and mild patient populations at age*FEV1% products of approximately 1000 and 1600 [10]. The distribution of the age*FEV1% predicted product in this current less-restricted patient population demonstrated a rightward shift of the distribution compared to the earlier data as a result of including all genotypes (Figs. 1 and S2).

Brasfield scoring provides complementary lung disease information not reflected in lung physiology studies as reflected in the residuals off the fitted linear regression model (S3 Fig.). Brasfield scoring of CF chest xrays has significant limitations including insensitivity to acute clinical changes and to disease in smaller airways [13]. However, the wide availability of chest xrays and the anatomic information provided by the studies outweigh these limitations for these phenotyping studies.

### Classification

The classes generated using all variables in the training cohort were associated with higher classification error rates. A supervised Random Forest analysis using the PAM clustering classes k = 3, k = 4, k = 5 and k = 6 demonstrated that eight variables i.e. FEV1% predicted, FVC% predicted, gender, age, height, weight, BMI and the age*FEV1% predicted product were consistently the most important variables in the classification and resulted in the lowest out of bag error rates. (Fig. 2A) This pattern was very similar with the k = 3, k = 4, and k = 6 classifications. Using a dimension reduction strategy, the proximity matrix was recalculated using these same eight variables from the training dataset. The PAM classifications based on the new identity matrix demonstrated lower classification errors (confusion matrix) and smaller out of bag error rates for k = 4, and k = 5 of 8.0%, and 13.3% respectively (Fig. 2B). Visualization of the classes using multidimensional scaling (MDS) confirmed good separation of the classes using this strategy. (Fig. 3)

### Multidimensional Clinical Phenotype (MDCP) Descriptions

**Fig 4:**
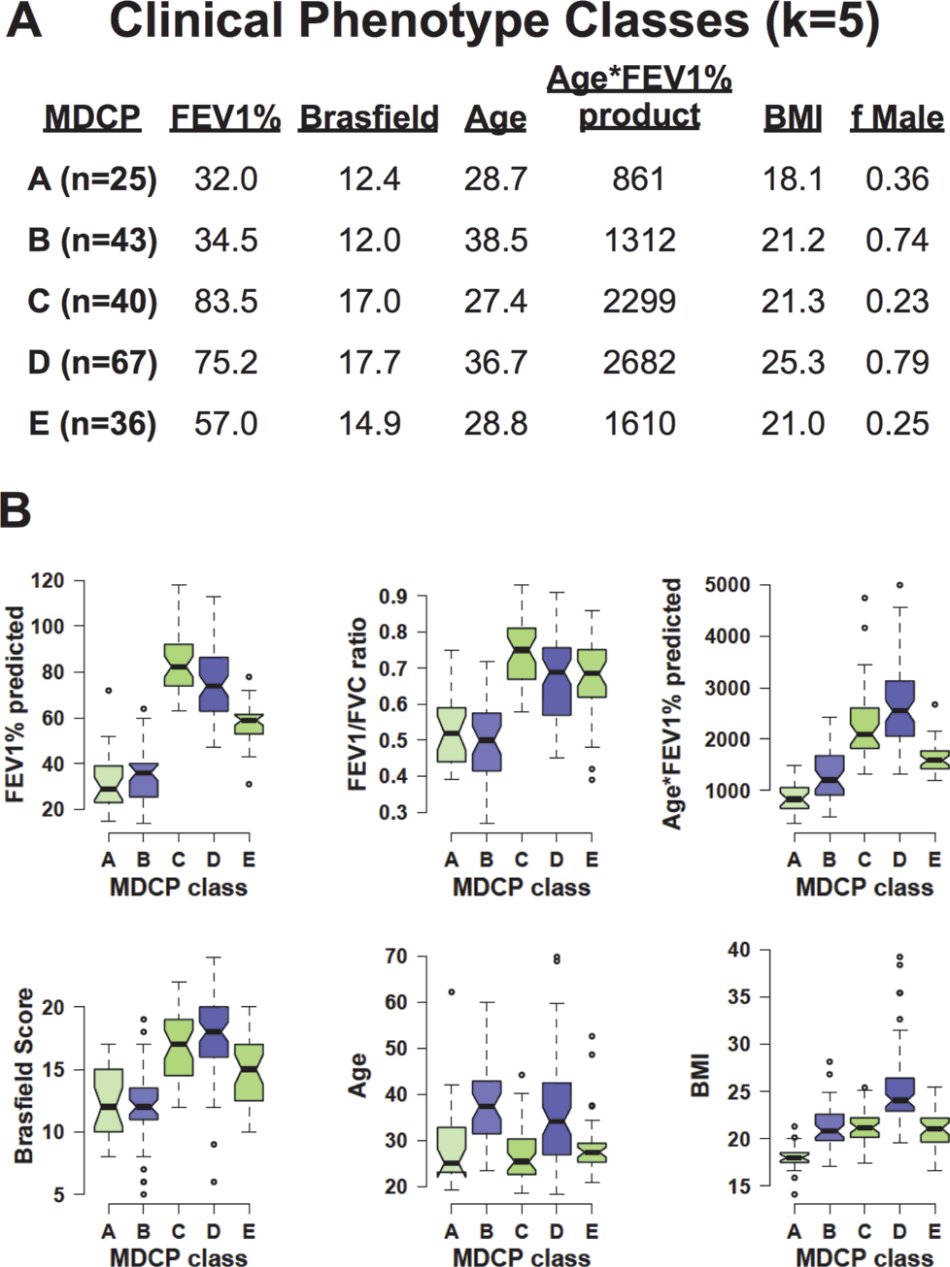
Multidimensional clinical phenotype (MDCP) descriptions. (A) Mean values of the FEV1%, total Brasfield chest xray score, age, age*FEV1% predicted, body mass index (BMI) and the fraction of males in each phenotype are shown. (B) Notchplots of the FEV1% predicted, FEV1 FVC ratio, age*FEV1% predicted, Brasfield chest xray score, age and BMI are plotted. The color of the notchplot boxes indicate the proportion of males in each class ranging from 0.0 male (green),. 5 male (white) to 1.0 male (blue).

Interpretation of the classes is shown in Fig. 4 (k = 5) and S4 Fig. (k = 4). In Fig. 4, the means and distribution of the age, BMI, FEV1%, Brasfield score, Age*FEV1% product and fraction of males in each phenotype (k = 5) is shown. This clustering strategy identified a group with the lowest age*FEV1% product (Class A). This class was also characterized by low FEV1% predicted, Brasfield scores and not surprisingly lower BMI values. Another consistent phenotype across strategies was a group characterized by good nutrition, high age*FEV1% product and dominated by male subjects (Class D).

In addition, a class of older, predominantly male patients with mean age of 38 were identified in both strategies (Class B). This oldest class had intermediate lung disease health with age*FEV1% products in the 1300–1500 range, severe airway obstruction, and median BMI values. The remaining classes were female dominated classes that varied mostly in terms of lung function and lung disease risk (Classes C and E; k = 5).

### Correlation of phenotypes with nutritional and microbiological variables (Table 1)

Pancreatic sufficiency (PS) is associated with CFTR mutations that retain residual activity and is characterized by milder, less progressive lung disease. Multidimensional clinical phenotyping identified a much higher incidence of PS in Class D patients and much lower rates of PS in the phenotype with the lowest age*FEV1% predicted product (Class A, k = 4). Similarly, enrichment of CFTR Class IV, V, and VI mutations was found in Class D (k = 4 and k = 5), consistent with their good lung function and well nourished phenotype, while lower frequencies of these mutations were identified in higher risk Class A phenotypes. There was no enrichment of PS or CFTR Class IV,V or VI mutations in any of the quintile classes of FEV1% predicted and Age*FEV1% product of the same dataset (S1 and S2 Tables).

**Table 1:** Class versus nonclass comparison of traits for the k = 4 and k = 5 adult CF clinical phenotypes.

	MDCP Class	PS	CFTR Class IV, V VI	PA	Candida	PA/Candida	MSSA/ASP	Interpretation
**k = 4**	**A**	<.05 (p = .002)						Low A*FEV1
	**B**			2.7 (p = .04)				Low A*FEV1, older, male
	**C**		.27(p = .05)	.37 (p = .05)			4.2(p = .03)	Median A*FEV1, female
	**D**	5.0 (p = .0007)	3.2(p = .01)					High A*FEV1, older, male
**k = 5**	**A**		<.05(p = .04)	4.9 (p = .03)	4.4 (p = .005)	4.4(p = .01)		Low A*FEV1
	**B**			3.5 (p = .02)				Low A*FEV1, older, male
	**C**			.25 (p = .001)			4.5(p = .03)	Median A*FEV1, female
	**D**	5.2 (p = .0003)	3.4(p = .01)		.17 (p = .005)	.20(p = .01)		High A*FEV1, older, male
	**E**							Low A*FEV1, female

Entries are the significant Fisher exact test odds ratios (p-values corrected for multiple testing) for pancreatic sufficiency (PS), presence of at least one CFTR class IV, V and VI mutation, *Pseudomonas* spp (PA), *Candida* spp, as well as combinations of PA and *Candida* spp. (PA/Candida) and MSSA and *Aspergillus* spp. (MSSA/Asp).

There were important confirming correlations between the clinical phenotypes and clinical microbiology results. Chronic pseudomonas infections have long been associated with accelerated loss of lung function in cystic fibrosis patients [14,15]. Consistent with these findings, phenotypes with low lung function (Class A and B, k = 5 and Class B, k = 4) had much higher frequencies of PA airway infections while lower rates of PA in sputum were found in the median risk, female dominated classes. (Class C, k = 5 and k = 4). (Table 1)

*Candida* spp were seen more frequently in Class A (k = 5) phenotype and less frequently in the milder phenotypes such as Class D (k = 5). The study likely had too few subjects to demonstrate differences in frequencies of other bacterial species including MSSA, MRSA, *S maltophilia*, *Achromobacter* spp, *Aspergillus* spp, *Scedosporium* spp and mycobacteria in the clinical phenotypes. The quintiles of FEV1% and age*FEV1% predicted product showed similar patterns for both PA and *Candida* spp i.e. high frequencies in low lung function quantiles and lower frequencies in quintiles with milder lung function. (S2 Table)

The relative frequencies of PA, *Staphylococcal spp* (MRSA and MSSA), *Candida* spp as well as *Aspergillus* spp allowed preliminary associations of the phenotypes with multiple organisms. (Table 1 and S2) Although there were no strong correlations between PA *and* either MSSA, MRSA or *Aspergillus* spp, there was enrichment of the combination of PA and *Candida* spp in Class A (k = 5) with much lower frequencies of this pair in Class D (k = 5). Furthermore, there was very strong, statistically significant enrichment of *Aspergillus* spp with MSSA in Class C, (k = 4 and k = 5) but not with MRSA, other MDCPs or in the quintile Classes of FEV1% or age*FEV1% product.

### Stability of the clinical phenotypes

**Fig 5:**
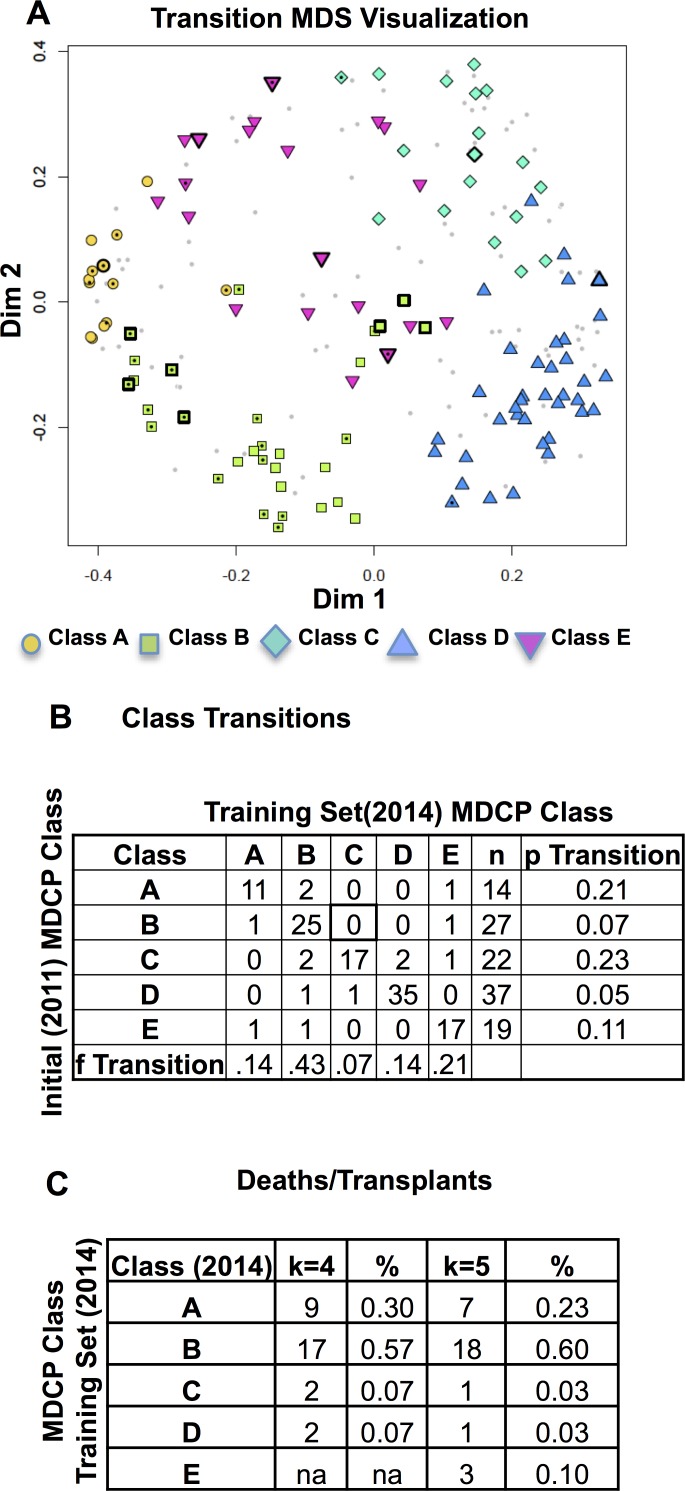
Clinical Phenotype Transitions. (A) The locations of subjects with deaths/transplants and class transitions are plotted on the MDS plot generated from the 2014 data (light gray dots). The colored points show the location and classes of patients present in both datasets. Positions with central black dots indicate subjects who died or had a lung transplant. Positions with thick black outlines of the points demonstrated class transitions. (B) Counts and frequencies of transitions between two phenotypes of subjects present in both cohorts (k = 5 phenotypes). The table shows the proportion of subjects leaving a particular clinical phenotype (p Transitions) and the fraction of total class transitions into a given clinical phenotype (f Transitions). (C) The counts of deaths or lung transplants in subjects represented in the 2014 cohort (k = 4 and k = 5) class phenotypes.

The availability of two datasets with the same variables separated by three years, allowed an assessment of the stability of the MDCPs. The subjects present in both datasets (n = 119) were evenly distributed in phenotypes (Fig. 5A). The most unstable phenotypes were Class A and the female-dominated, median age*FEV1% predicted product (Class C) phenotypes with just over 20% of the subjects transitioning to new classes over the three year period (Fig. 5B). In contrast, the most stable phenotypes were the older, male dominated phenotypes (Classes B and D) which had transition rates in the 5–7% over the same 3 year period.

The most common phenotypes that subjects moved into were Class B and Class E which were associated with 43 and 21 percent of the transitions respectively. The present study was too small to specifically identify which variables were driving these class transitions. Death and lung transplantation were rare in lower risk phenotypes (Class C and Class D, k = 4) with over 80% of these events occurring in phenotypes with lower age*FEV1% predicted product (Class A and B in Fig. 5C).

## Discussion

Generating clinical phenotypes facilitates assessment of disease prognosis, response to therapy, and provides insights into airway biology and disease pathophysiology [4,16]. We present a clinical phenotyping strategy that identified classes within a typical adult CF population with distinct characteristics in terms of age, gender, nutritional status, lung physiology and risk for CF lung disease. These phenotypes differ significantly from other strategies including quintile classes of FEV1% and the age*FEV1% product by identifying groups of patients that share multiple traits of the multi-system disease.

The described phenotyping strategy identified classes with predictable and validating but also unexpected traits. Not surprisingly, multi-dimensional clinical phenotyping identified classes with milder lung disease, PS and a higher frequency of CFTR Class IV, V, and VI mutations. The microbiology of CF is strongly driven by the airway microenvironment which is more closely reflected by lung physiology. PA is associated with more aggressive loss of lung function and so we anticipated higher frequencies of PA in higher risk groups and lower rates in the milder disease classes [14,15].

Ecological interactions between fungal and bacterial populations are well studied but the clinical implications are not fully appreciated [17,18]. In this dataset, we found two strong correlations between a) PA and *Candida* spp b) and MSSA and *Aspergillus* spp. with specific phenotypic classes that warrant further metagenomic and metabolomics studies. For instance, *Candida* spp and PA demonstrate a variety of specific ecological interactions that range from antagonistic to a fully symbiotic relationship which appears dependent contextually on the growth environment [18]. *In vitro* and *in vivo* studies support synergistic interactions between MSSA and *Aspergillus* spp [19,20]. These symbiotic relationships likely promote the unstable phenotype seen in Class C subjects.

Clinical scoring systems have seen significant use in CF over the past 50 years [16]. These scoring systems focus on different aspects of the disease including overall disease progression, physiology, effects of short-term intervention, radiography, nutrition and more recently health related quality of life. For example, the modified Shwachman/Kulcyski score and the Taussig-NIH scoring systems are extensively-used, validated systems that consider radiography, physical, nutritional and historical findings but focus predominantly on pediatric CF populations [21,22,23]. Huang et al. and later Matouk developed a clinical scoring system relevant to adult CF populations that incorporates physiological, radiographic and clinical parameters and was most useful for tracking patients longitudinally [24,25]. In addition to these multivariable clinical scoring systems, others have a narrower focus of this multi-systemic disease assessing limited aspects such as radiography, nutrition, or quality of life.

An approach similar to this current study was taken by Hafen et. al. in a pediatric population which relied on semi-quantitative provider assessments of disease severity to identify the clinical variables critical in classifying the subjects [26]. The current approach was not designed to develop a new scoring system for adult CF but to generate clinical phenotypes based on stable demographic and frequently measured clinical, radiographic, nutritional, physiological and microbiological data using a minimal amount of subjective data.

Limitations of the current study include the regional nature of the study population and its small size which did not allow confirmation of well-known correlations between lung physiology and microbiology including the association of MRSA strains or the role of rarer specific CFTR mutations with lung function. Validation of the phenotypes in a larger and regionally distinct adult CF populations as well as establishing their clinical and biological relevance by correlating phenotypes with metagenomic, metabolomic and proteomic data or responses to specific therapies remains a priority.

Multidimensional clinical phenotyping offers opportunities to characterize a multisystemic disease with interacting phenotypes that is not possible with simpler strategies. The approach in this study allows complex data of nearly any type-categorical, numerical or ordinal data. The phenotypes generated were clinically relevant confirming not only published findings but also suggested new microbiological interactions worthy of further study. This approach generates phenotypes that will allow further studies on CF long-term survivors, patients at high risk for disease progression, and differences in gender outcomes.

## Supporting Information

S1 Fig Comparison of the Excluded versus Training adult CF patient cohorts. (A) Notchplots comparing the FEV1% predicted, FEV1/FVC ratio, Age*FEV1% product, Brasfield chest Xray scores, age and BMI. (B) Proportions of pancreatic sufficiency (PS), fraction of male subjects (f Male) and presence of two CFTR Class I, II and III mutations. The Fisher exact test odds ratio with the p-value are presented. Notchplots demonstrate the median, 25 percentile, 75 percentile and outlier values. The extending bars demonstrate the span between 1.5 x interquartile range above and below the median. Nonoverlapping notched areas likely represent significant differences between two groups.
(TIFF)

S2 Fig Scatterplot of the Age*FEV1% predicted product versus FEV1% predicted of the training set. Shown are the positions of the subjects in each clinical phenotype (k = 4). The median values (arrowheads on axes) as well as the 25^th^ and 75^th^ percentile (dotted gray lines) are shown for the FEV1% predicted and Age*FEV1% predicted product.
(TIFF)

S3 Fig Scatterplot of Brasfield chest xray score versus FEV1% predicted. Shown are the positions of subjects in each clinical phenotype (k = 4). Also shown is the linear regression line and the equation model.
(TIFF)

S4 Fig Multidimensional clinical phenotypes for the k = 4 strategy. Mean values of the FEV1%, total Brasfield chest xray score, age, age*FEV1% predicted, body mass index (BMI) and the fraction of males in each phenotype are shown. (B) Notchplots of the FEV1% predicted, FEV1 FVC ratio, age*FEV1% predicted, Brasfield chest xray score, age and BMI are plotted. The color of the notchplot boxes indicate the proportion of males in each class ranging from 0.0 male (green),. 5 male (white) to 1.0 male (blue).
(TIFF)

S1 Table Characterization of the quintile classes of FEV1% predicted and the age*FEV1% product of the training data set. The table shows the mean values of the class FEV1%, FVC%, Brasfield chest xray score, age, age*FEV1% product, body mass index (BMI) and the fraction of male subjects.
(PDF)

S2 Table Class versus nonclass comparison of traits for FEV1% predicted and Age*FEV1% predicted product quintiles of adult CF subjects. Entries are the significant Fisher exact test odds ratios (p-values corrected for multiple testing) for pancreatic sufficiency (PS), presence of at least one CFTR class IV, V and VI mutation, *Pseudomonas* spp (PA), *Candida* spp, as well as the combination of PA and *Candida* spp. (PA/Candida).
(PDF)
