# Supplementary figures and images for: Multidimensional Clinical Phenotyping of an Adult Cystic Fibrosis Patient Population
Source: PLoS One. 2015 Mar 30;10(3):e0122705. doi: 10.1371/journal.pone.0122705 (PMC4378917; doi:10.1371/journal.pone.0122705)

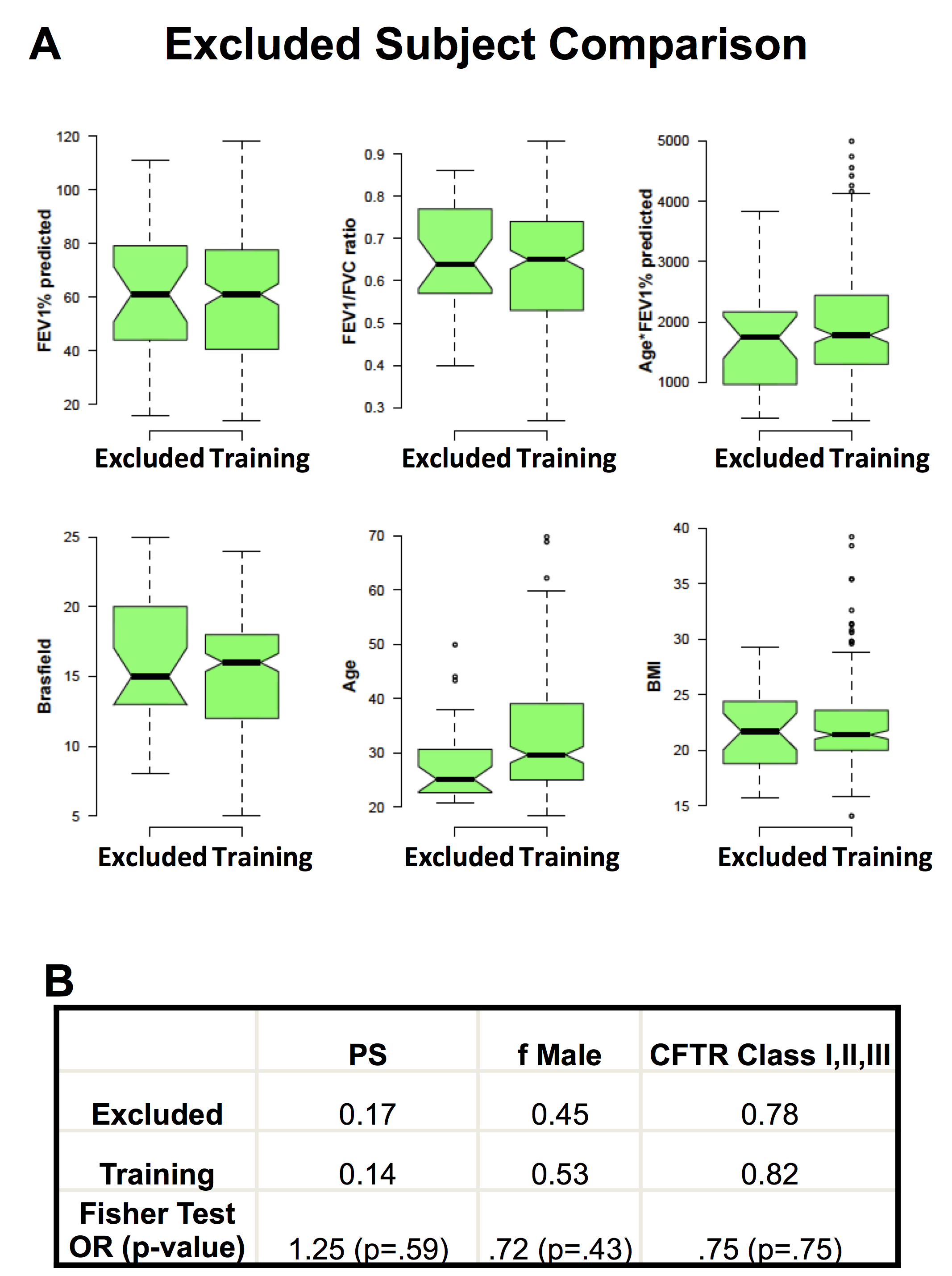

Supplement: S1 Fig — (A) Notchplots comparing the FEV1% predicted, FEV1/FVC ratio, Age*FEV1% product, Brasfield chest Xray scores, age and BMI. (B) Proportions of pancreatic sufficiency (PS), fraction of male subjects (f Male) and presence of two CFTR Class I, II and III mutations. The Fisher exact test odds ratio with the p-value are presented. Notchplots demonstrate the median, 25 percentile, 75 percentile and outlier values. The extending bars demonstrate the span between 1.5 x interquartile range above and below the median. Nonoverlapping notched areas likely represent significant differences between two groups. (TIFF) [file pone.0122705.s001.tiff]

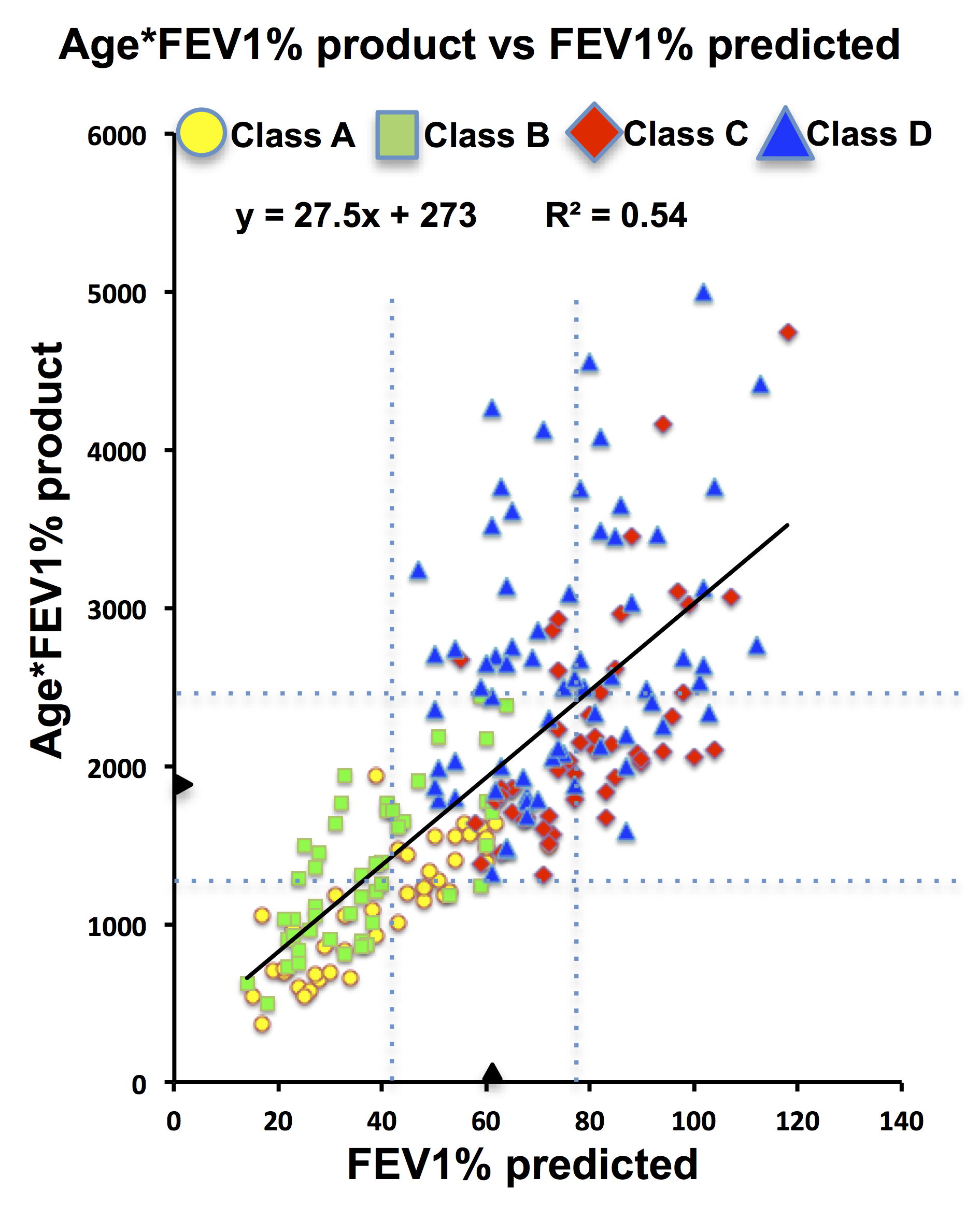

Supplement: S2 Fig — Shown are the positions of the subjects in each clinical phenotype (k = 4). The median values (arrowheads on axes) as well as the 25th and 75th percentile (dotted gray lines) are shown for the FEV1% predicted and Age*FEV1% predicted product. (TIFF) [file pone.0122705.s002.tiff]

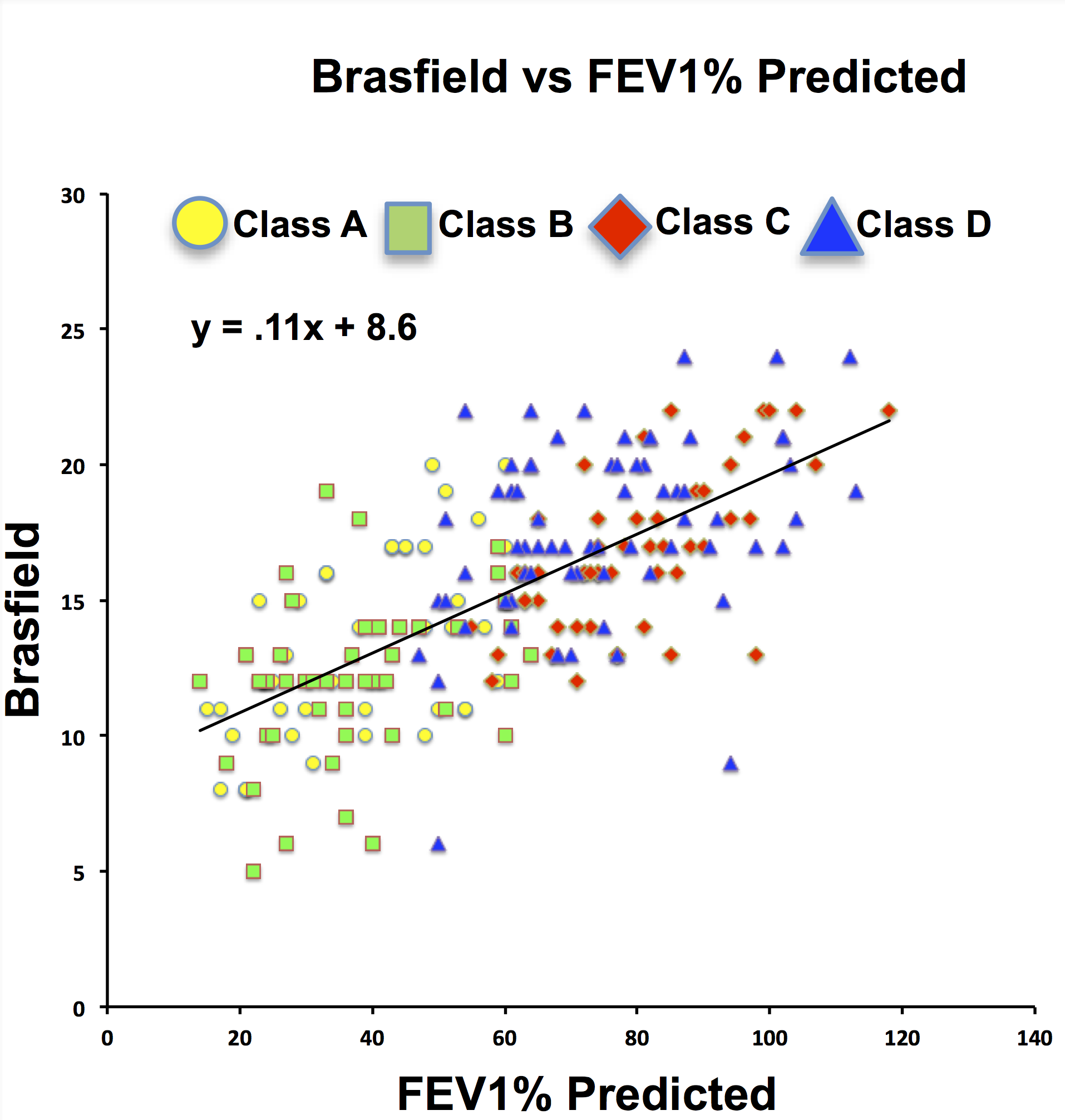

Supplement: S3 Fig — Shown are the positions of subjects in each clinical phenotype (k = 4). Also shown is the linear regression line and the equation model. (TIFF) [file pone.0122705.s003.tiff]

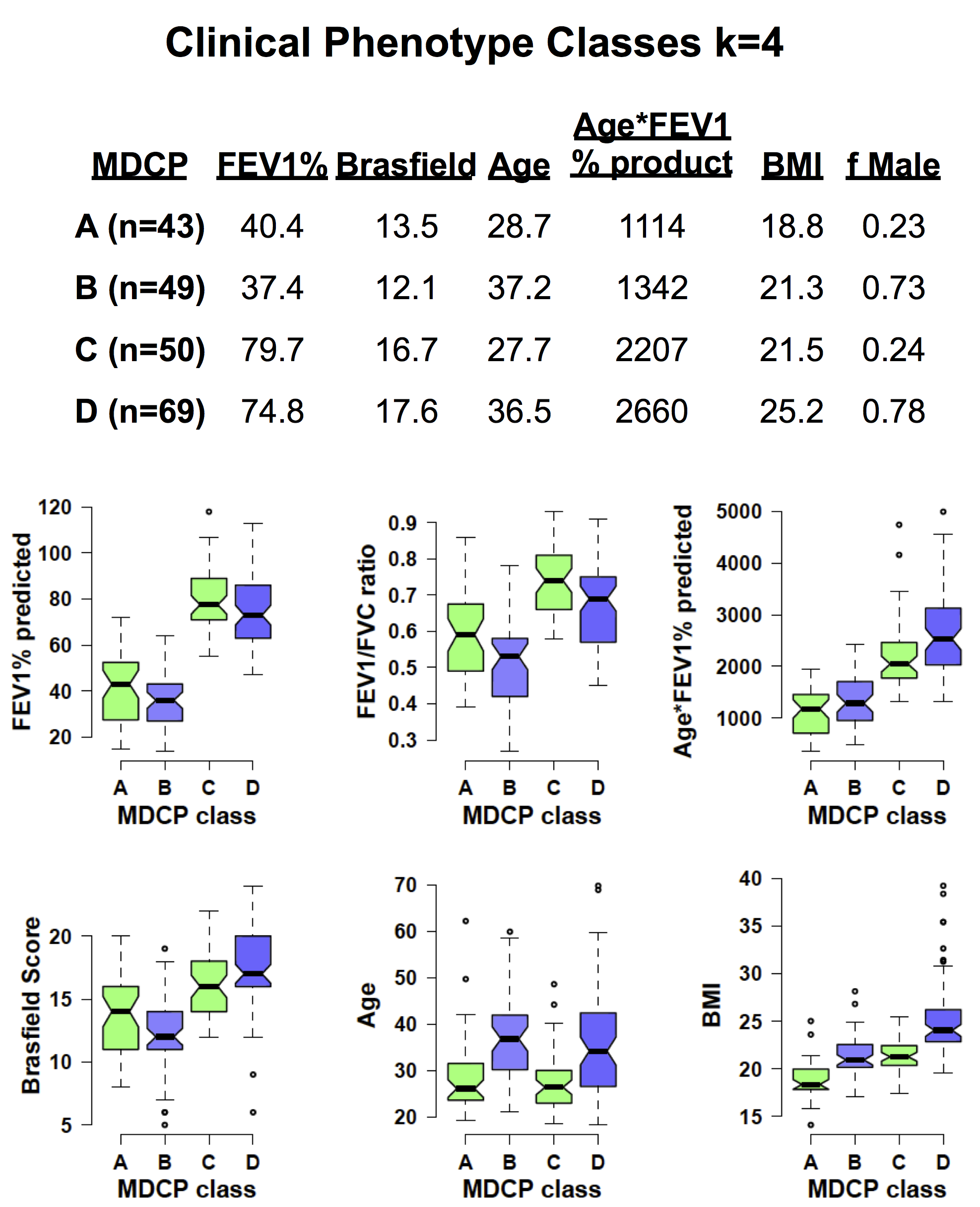

Supplement: S4 Fig — Mean values of the FEV1%, total Brasfield chest xray score, age, age*FEV1% predicted, body mass index (BMI) and the fraction of males in each phenotype are shown. (B) Notchplots of the FEV1% predicted, FEV1 FVC ratio, age*FEV1% predicted, Brasfield chest xray score, age and BMI are plotted. The color of the notchplot boxes indicate the proportion of males in each class ranging from 0.0 male (green),. 5 male (white) to 1.0 male (blue). (TIFF) [file pone.0122705.s004.tiff]
