# Supplementary material for: Multidimensional Clinical Phenotyping of an Adult Cystic Fibrosis Patient Population
Source: PLoS One. 2015 Mar 30;10(3):e0122705. doi: 10.1371/journal.pone.0122705 (PMC4378917; doi:10.1371/journal.pone.0122705)
Supplement: S1 Table — The table shows the mean values of the class FEV1%, FVC%, Brasfield chest xray score, age, age*FEV1% product, body mass index (BMI) and the fraction of male subjects. (PDF) [file pone.0122705.s005.pdf]

	FEV1%	FVC%	Brasfield	Age	Age*FEV1% product	BMI	f Male
**FEV1% quintile**							
**1 (n=42)**	26.5	49.2	11.4	36.3	948	20.1	0.66
**2 (n=42)**	46.3	69.1	13.8	34.3	1589	21.3	0.46
**3 (n=42)**	61.6	81.1	16.0	33.8	2081	22.6	0.58
**4 (n=43)**	75.0	90.2	16.8	31.2	2351	23.2	0.63
**5 (n=42)**	94.5	103	18.7	29.0	2753	23.4	0.30
**A*FEV1 prod quintile**							
**1 (n=43)**	28.5	49.3	11.6	31.4	854	19.5	0.60
**2 (n=42)**	52.3	70.9	14.6	29.6	1419	21.6	0.43
**3 (n=42)**	61.1	82.2	15.0	31.3	1801	22.3	0.55
**4 (n=42)**	79.9	94.0	17.3	29.6	2279	22.4	0.50
**5 (n=42)**	80.4	95.0	17.8	43.1	3331	24.8	0.57
