# Supplementary material for: Multidimensional Clinical Phenotyping of an Adult Cystic Fibrosis Patient Population
Source: PLoS One. 2015 Mar 30;10(3):e0122705. doi: 10.1371/journal.pone.0122705 (PMC4378917; doi:10.1371/journal.pone.0122705)
Supplement: S2 Table — Entries are the significant Fisher exact test odds ratios (p-values corrected for multiple testing) for pancreatic sufficiency (PS), presence of at least one CFTR class IV, V and VI mutation, Pseudomonas spp (PA), Candida spp, as well as the combination of PA and Candida spp. (PA/Candida). (PDF) [file pone.0122705.s006.pdf]

	quintile	PS	CFTR Class IV,V,VI	PA	Candida	PA/Candida
**FEV1%**	**1**			2.8 (p=.04)	4.7 (p=.001)	5.2 (p=.0005)
	**2**					
	**3**					
	**4**			.38 (p=.03)		
	**5**			.33 (p=.01)	.11 (p=.02)	
	**quintile**					
**Age*FEV1%**	**1**				5.7 (p=.0001)	6.4 (p=.0001)
**product**	**2**					
	**3**					
	**4**			.28 (p=.002)		
	**5**				.10 (p=.01)	.12 (p=.03)
